# The Relationship Between Physical Activity and Mobile Phone Addiction Among Adolescents: A Cross-Lagged Study

**DOI:** 10.3390/bs16091557

**Published:** 2026-09-02

**Authors:** Hui Wang, Yongguan Dai

**Affiliations:** 1 School of Physical Education, Jiangxi Normal University, Nanchang 330022, China; 2 School of Physical Education, Guangzhou University, Guangzhou 510006, China

**Keywords:** physical activity, problematic smartphone use, adolescents, random-intercept cross-lagged panel model, cross-lagged panel model, longitudinal study, within-person dynamics

## Abstract

Insufficient physical activity (PA) and problematic smartphone use (PSU) frequently co-occur during adolescence, but whether their prospective association reflects stable differences between adolescents or within-person fluctuations remains unclear. This three-wave longitudinal study examined reciprocal prospective associations between PA and PSU in 1015 Chinese adolescents aged 12–18 years across 12 months, with assessments separated by approximately six months. PA was assessed using the Physical Activity Rating Scale-3, and PSU was assessed using the Smartphone Addiction Scale–Short Version. A random-intercept cross-lagged panel model (RI-CLPM) was specified as the primary analysis, with a conventional cross-lagged panel model (CLPM) estimated secondarily for comparison with previous longitudinal research. In the RI-CLPM, the stable between-person components of PA and PSU were negatively associated (β = −0.681, *p* = 0.004). At the within-person level, higher-than-usual PA was prospectively associated with lower-than-usual PSU at the subsequent wave (β = −0.117 and −0.108), whereas higher-than-usual PSU was prospectively associated with lower-than-usual subsequent PA (β = −0.257 and −0.189); all four cross-lagged paths were statistically significant. The secondary CLPM showed reciprocal negative cross-lagged associations in the same directions. By separating stable between-person differences from within-person deviations, this study extends longitudinal evidence on the PA–PSU relationship and indicates that the two behaviors are prospectively coupled within adolescents over time. These findings provide a basis for future research and intervention trials that consider PA and problematic smartphone behavior jointly, while causal mechanisms remain to be established.

## 1. Introduction

In recent years, insufficient physical activity (PA) and problematic smartphone use (PSU) have emerged as concurrent behavioral concerns among adolescents, with potential implications for physical, psychological, and social development. A WHO-led pooled analysis showed that approximately 81% of school-going adolescents aged 11–17 years worldwide did not meet the recommended average of at least 60 min of moderate-to-vigorous physical activity (MVPA) per day, with insufficient PA reported in approximately 85% of girls and 78% of boys (Bull et al., 2020; Sahu et al., 2019; Guthold et al., 2019; van Sluijs et al., 2021). Meanwhile, smartphones have become increasingly embedded in adolescents’ learning, social interaction, information seeking, and entertainment, raising concerns about PSU and its associations with mental health, well-being, and daily functioning (Fischer-Grote et al., 2019, 2021; Harris et al., 2020). A systematic review and meta-analysis estimated a median PSU prevalence of 23.3% among children and young people (Sohn et al., 2019), while a broader meta-analysis covering studies published between 2012 and 2022 reported a pooled prevalence of 37.1% across age groups (Lu et al., 2024).

In China, the number of underage internet users reached 196 million in 2023, with an internet penetration rate of 97.3% (Du et al., 2024). At the same time, PA levels among Chinese adolescents remain low. Using nationally collected data from 2017 to 2019, Guo et al. (2024) reported that, in 2019, only 17.61% of middle school students and 12.56% of high school students met recommended PA levels. Longitudinal evidence has also begun to examine the temporal association between PA and PSU. In a three-wave study of 2131 adolescents, Zhao et al. (2024) reported reciprocal negative prospective associations between PA and PSU across one-year intervals.

Previous adolescent research has consistently identified an inverse association between PA and PSU, raising the possibility that higher PA may precede lower subsequent PSU (Ji et al., 2024). One possible explanation is time displacement. Because adolescents have limited discretionary time, greater participation in sports, outdoor recreation, and other physically active pursuits may reduce the time available for smartphone-based entertainment, gaming, and social networking. Prospective studies in adolescent populations have provided some support for this possibility, although the extent of displacement may vary across activities and time scales (Hu et al., 2026c; Huang et al., 2026). Self-regulation may also be relevant. Self-control has been associated with activity-related behaviors and PSU among adolescents and has been proposed as a potential pathway linking PA with subsequent smartphone-use patterns (Hu et al., 2025b; Yang et al., 2026; Yuan et al., 2025). These explanations remain theoretically plausible rather than mechanisms established by the available longitudinal evidence.

The reverse direction is also plausible. Higher PSU may precede lower subsequent PA when smartphone use competes with time otherwise available for exercise, outdoor recreation, or active social participation. Smartphone use may also coincide with disruptions in daily routines that are relevant to PA. Studies using intensive or sensor-based assessments have linked greater smartphone use with less exercise, fewer steps, and more sedentary time among adolescents (Wan et al., 2025; X. Yue et al., 2025), while longitudinal evidence has associated problematic mobile phone use with subsequent changes in sleep-related behavior and PA (Hu et al., 2025a, 2026a). These findings support examining PA and PSU as potentially reciprocal behavioral processes rather than assuming a unidirectional relationship.

Despite growing interest in this relationship, its temporal structure remains insufficiently understood. Much of the available evidence is cross-sectional, limiting conclusions about temporal ordering, and relatively few adolescent studies have examined reciprocal associations across multiple measurement occasions. More importantly, conventional longitudinal analyses may not distinguish differences between adolescents from fluctuations occurring within the same adolescent over time. A negative association observed between adolescents does not necessarily imply that, when an individual adolescent becomes more physically active than usual, that adolescent will subsequently report lower-than-usual PSU. Distinguishing these levels of variation is therefore necessary when the research question concerns intraindividual change.

The conventional Cross-Lagged Panel Model (CLPM) is widely used to estimate prospective associations between repeatedly measured constructs. By incorporating autoregressive and cross-lagged paths, the CLPM assesses whether an individual’s relative standing on one construct at an earlier occasion is associated with their relative standing on another construct at a later occasion after accounting for prior levels of the outcome. However, conventional CLPM estimates do not separate stable between-person differences from time-varying within-person deviations (Hamaker et al., 2015). Cross-lagged coefficients may therefore reflect a mixture of enduring differences between adolescents and occasion-specific fluctuations within adolescents, which limits their interpretation when the primary interest lies in within-person processes.

The Random Intercept Cross-Lagged Panel Model (RI-CLPM) addresses this limitation by separating stable between-person differences from time-specific within-person deviations through construct-specific random intercepts (Mulder & Hamaker, 2021; Orth et al., 2021). At the between-person level, the association between the random intercepts indicates whether adolescents who are generally more physically active across the study period also tend to report lower overall levels of PSU. At the within-person level, cross-lagged paths indicate whether a deviation from an adolescent’s own expected level of PA at one measurement occasion is prospectively associated with a subsequent deviation in PSU, and vice versa. The autoregressive parameters in the RI-CLPM similarly represent the persistence of within-person deviations over time rather than the rank-order stability estimated in the conventional CLPM. These parameters describe prospective associations and do not, by themselves, establish causal effects.

The present study used a three-wave longitudinal design to examine reciprocal prospective associations between PA and PSU among Chinese adolescents. Because the primary research question concerned within-person dynamics after accounting for stable between-person differences, the RI-CLPM was specified as the primary analytical model. A conventional CLPM was also estimated as a secondary model to facilitate comparison with previous longitudinal research. The following hypotheses were examined:

**H1.**  *At the between-person level, stable individual differences in PA are negatively associated with stable individual differences in PSU.*

**H2.**  *At the within-person level, higher-than-usual PA at one measurement occasion is prospectively associated with lower-than-usual PSU at the subsequent occasion.*

**H3.**  *At the within-person level, higher-than-usual PSU at one measurement occasion is prospectively associated with lower-than-usual PA at the subsequent occasion.*

## 2. Methods

### 2.1. Participants and Procedure

Participants were recruited through convenience sampling from six secondary schools, including junior and senior high schools, in Jiangxi Province, China. A three-wave longitudinal design spanning 12 months was employed, with data collected in May 2025 (Time 1, T1), November 2025 (Time 2, T2), and May 2026 (Time 3, T3). An approximately six-month interval was selected between consecutive assessments based on previous longitudinal studies that used similar follow-up periods to examine changes in behavioral and psychological outcomes over time (Hu et al., 2026b; Y. Yue et al., 2026).

At baseline, students in Grades 9 and 12 were not recruited because these students were preparing for the senior high school entrance examination and the National College Entrance Examination, respectively, and were also approaching graduation, which could interfere with continued school-based follow-up. Eligible participants at T1 were therefore students enrolled in Grades 7, 8, 10, and 11. Students in Grades 8 and 11 at baseline remained in the cohort as they progressed into Grades 9 and 12 during the follow-up period.

Questionnaires were administered collectively during regular school hours by trained research assistants using standardized instructions. Ethical approval was obtained from the Ethics Committee of Jiangxi Normal University, and all procedures were conducted in accordance with the Declaration of Helsinki. Before data collection, written informed consent was obtained from participants aged 18 years and from the parents or legal guardians of participants younger than 18 years. Written assent was also obtained from all participating minors. Participants were informed that participation was voluntary, that their responses would remain confidential, and that they could withdraw from the study at any time without penalty. To link questionnaires across waves while preserving anonymity, each participant generated a unique identification code. The same measures of PA and PSU were administered at all three assessments.

**Figure 1:**
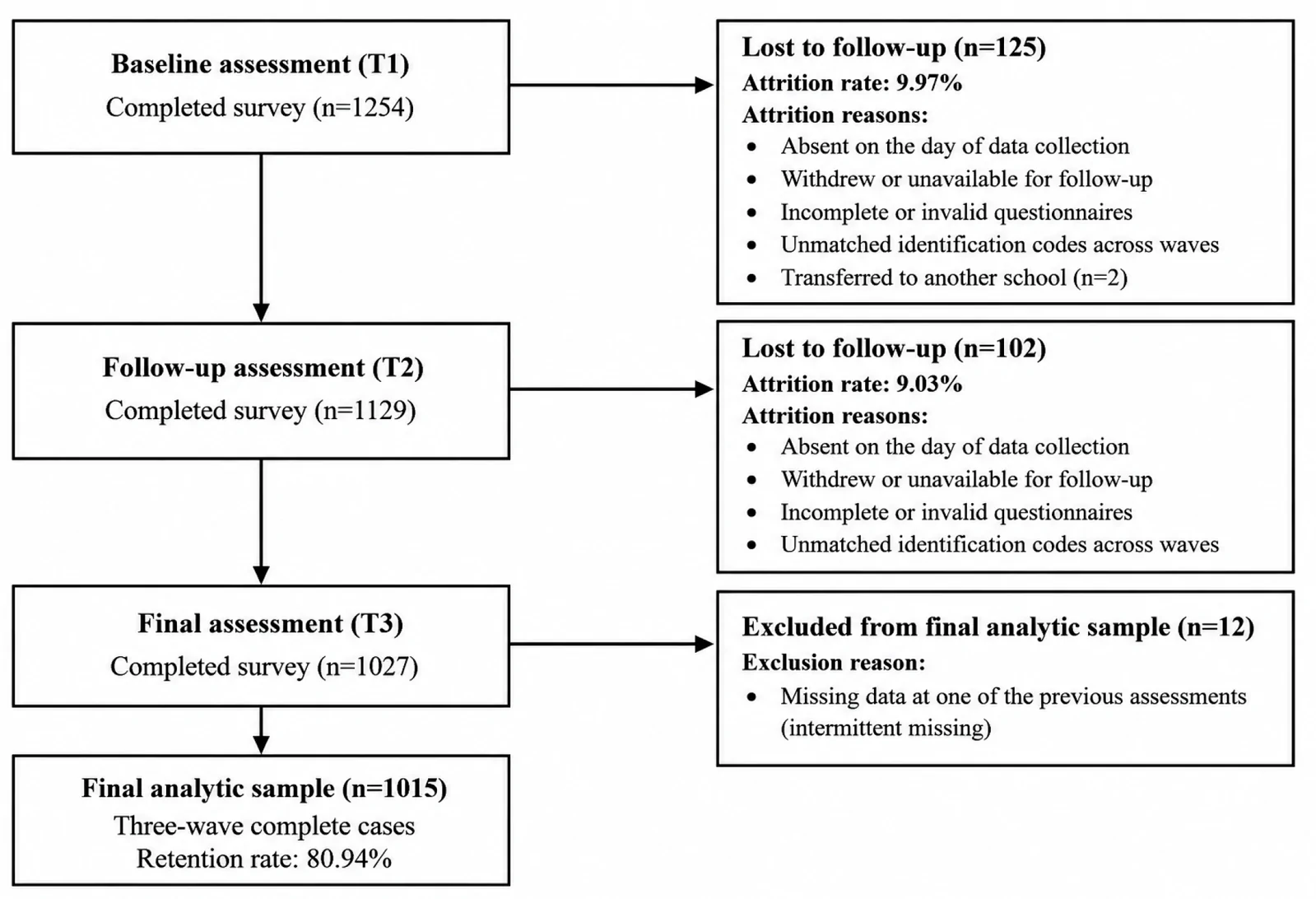
Flowchart of participant recruitment, follow-up, attrition, and inclusion in the final analytic sample.

At T1, 1254 adolescents provided valid baseline questionnaires. At T2, 1129 participants completed the follow-up assessment, corresponding to an attrition rate of 9.97% from baseline. At T3, 1027 adolescents provided valid data, representing a further attrition rate of 9.03% from T2. Participant loss across the follow-up period was mainly attributable to absence on the day of data collection, withdrawal or inability to continue participation, incomplete or invalid questionnaires, unmatched identification codes across waves, and school transfer; only two participants were lost because of school transfer during the follow-up period. Among the 1027 adolescents assessed at T3, 1015 had successfully matched records and complete data across all three waves, whereas 12 had missed one of the previous assessments. The detailed participant flow and reasons for attrition are presented in Figure 1.

For the longitudinal analyses, the analytic sample was restricted to adolescents with successfully matched data across all three assessment waves. This complete-case approach was selected to maintain a consistent cohort across T1, T2, and T3 and to ensure that the CLPM and RI-CLPM estimates were based on the same participants throughout the longitudinal comparisons. Accordingly, 1015 adolescents constituted the final analytic sample, corresponding to a three-wave retention rate of 80.94% and a cumulative attrition rate of 19.06%.

Baseline attrition analyses compared adolescents included in the final analytic sample with those who did not complete all three assessments. Comparisons were conducted for age, sex, grade, baseline PA, and baseline PSU, with both statistical significance and effect-size estimates considered when evaluating potential differences between groups. Detailed results are provided in Supplementary Table S1.

The final analytic sample comprised 425 boys (41.87%) and 590 girls (58.13%). At baseline, participants ranged in age from 12 to 18 years, with a mean age of 14.86 years (SD = 1.61). The sample included 280 students in Grade 7 (27.59%), 270 in Grade 8 (26.60%), 245 in Grade 10 (24.14%), and 220 in Grade 11 (21.67%).

### 2.2. Measures

#### 2.2.1. Physical Activity (PA)

PA was assessed at each measurement wave using the Physical Activity Rating Scale-3 (PARS-3), developed by Liang (1994). The PARS-3 is a three-item instrument designed to evaluate physical activity during the preceding month across three components: exercise intensity, duration per session, and exercise frequency. This scale has been widely used to assess physical activity in Chinese populations and has demonstrated satisfactory psychometric properties across different age groups (Hu et al., 2026b, 2026d; W. Zhang et al., 2026). Following the standard scoring procedure, the total PA score was calculated using the following formula: PA score = intensity × (duration − 1) × frequency. Total scores range from 0 to 100, with higher scores indicating higher levels of physical activity. The scale demonstrated acceptable internal consistency across the three waves, with Cronbach’s α coefficients of 0.733 at T1, 0.728 at T2, and 0.742 at T3.

#### 2.2.2. Problematic Smartphone Use (PSU)

PSU was assessed at each wave using the Smartphone Addiction Scale–Short Version (SAS-SV), originally developed and validated by Kwon et al. (2013) for adolescents. The SAS-SV is a 10-item self-report measure assessing the severity of problematic smartphone-use symptoms, including daily-life disturbance, withdrawal, overuse, and tolerance. Although the scale was originally developed in 2013, subsequent psychometric evaluations have continued to support its use in adolescent populations. In particular, the Chinese version has demonstrated satisfactory reliability and validity among Chinese children and adolescents (T. Cheung et al., 2019; Yang et al., 2026), supporting its applicability to the population examined in the present study. Participants rated each item on a six-point Likert scale ranging from 1 (strongly disagree) to 6 (strongly agree). Item scores were summed to obtain a total score ranging from 10 to 60, with higher scores indicating greater severity of PSU symptoms. The total score was treated as a continuous measure rather than as an indicator of a clinical diagnosis. In the present sample, the SAS-SV demonstrated good internal consistency across all three waves, with Cronbach’s α coefficients of 0.867 at T1, 0.865 at T2, and 0.864 at T3.

### 2.3. Statistical Analysis

All statistical analyses were conducted using SPSS version 26.0 and Mplus version 8.3. Preliminary analyses included data screening, descriptive statistics, distributional assessment, and Pearson correlation analyses. Means, standard deviations, skewness, and kurtosis were calculated for PA and PSU at each measurement wave. Given the composite scoring structure of the PARS-3, the proportion of zero PA scores was also examined. Internal consistency was evaluated for the SAS-SV but not for the PARS-3, which was treated as a composite exercise-volume index rather than a reflective scale.

Potential selective attrition was examined by comparing adolescents included in the final three-wave analytic sample with those who did not complete all three assessments. Independent-samples *t* tests were used for baseline age, PA, and PSU, whereas chi-square tests were used for sex and grade. Effect-size estimates were considered alongside statistical significance, and detailed attrition analyses are reported in Supplementary Table S1. For the longitudinal models, analyses were conducted using the 1015 adolescents with successfully matched data across all three waves. This complete-case strategy was adopted to retain an identical analytic cohort across T1, T2, and T3 and across the CLPM and RI-CLPM analyses. Because this approach excludes information from participants with incomplete follow-up, the potential implications of complete-case analysis are considered in the Limitations section.

Longitudinal measurement invariance was evaluated for the SAS-SV across T1, T2, and T3 before estimating the structural models. Configural, metric, and scalar invariance models were tested sequentially. Longitudinal invariance was considered supported when increasingly restrictive models showed changes of no more than 0.010 in the Comparative Fit Index (|ΔCFI| ≤ 0.010) and no more than 0.015 in the Root Mean Square Error of Approximation (|ΔRMSEA| ≤ 0.015) (Chen, 2007; G. W. Cheung & Rensvold, 2002). Because the intensity, duration, and frequency components of the PARS-3 jointly define a composite exercise-volume score rather than interchangeable reflective indicators of a latent construct, reflective longitudinal measurement-invariance testing was not conducted for the PARS-3.

The RI-CLPM was specified as the primary longitudinal model because the principal research question concerned reciprocal associations between time-specific within-person deviations in PA and PSU after accounting for stable between-person differences. The model was estimated using the observed PARS-3 and SAS-SV total scores at each wave. Separate random-intercept factors were specified for PA and PSU, with their loadings on the three repeated measures fixed to 1. Time-specific within-person latent components were defined for each repeated measure with unit loadings, and the residual variances of the observed repeated measures were fixed to zero. The two random intercepts were allowed to covary, thereby representing the stable between-person association between PA and PSU across the study period.

Within the within-person part of the RI-CLPM, autoregressive and reciprocal cross-lagged paths were freely estimated across the two six-month intervals. Autoregressive paths represented carry-over in deviations from an adolescent’s own expected level, whereas cross-lagged paths represented whether a deviation in one behavior at a given wave was prospectively associated with a subsequent deviation in the other behavior. Within-wave covariances between the time-specific PA and PSU components were also freely estimated at T1, T2, and T3. Model estimation was inspected for convergence problems, inadmissible solutions, and negative freely estimated variance components.

A conventional CLPM was estimated as a secondary analysis to facilitate comparison with previous longitudinal studies. Autoregressive paths were specified between adjacent waves for both PA and PSU, together with reciprocal cross-lagged paths from PA to subsequent PSU and from PSU to subsequent PA. PA and PSU were allowed to covary at T1, and their residuals were allowed to covary within T2 and T3. Because the conventional CLPM does not separate stable between-person differences from within-person fluctuations, its parameters were interpreted separately from the within-person estimates obtained from the RI-CLPM.

Both structural models were estimated using maximum likelihood estimation with robust standard errors (MLR), which provides standard errors and test statistics that are relatively robust to departures from multivariate normality. Model fit was evaluated using the chi-square statistic (χ^2^), Comparative Fit Index (CFI), Tucker–Lewis Index (TLI), Root Mean Square Error of Approximation (RMSEA), and Standardized Root Mean Square Residual (SRMR). CFI and TLI values of at least 0.90 together with RMSEA and SRMR values of no more than 0.08 were considered indicative of acceptable fit, whereas CFI and TLI values of at least 0.95 and RMSEA and SRMR values of no more than 0.05 were considered indicative of good fit (Bentler & Bonett, 1980; Browne & Cudeck, 1992). Because chi-square is sensitive to sample size, model evaluation was based on the overall pattern of fit indices rather than on the chi-square statistic alone. For the RI-CLPM, fit indices were interpreted cautiously because the model had only one degree of freedom.

Unstandardized estimates (B), robust standard errors, 95% confidence intervals, and STDYX-standardized coefficients (β) were reported for the RI-CLPM parameters. Coefficients of determination (R^2^) were additionally reported for the endogenous within-person components. Statistical significance was evaluated using two-tailed tests with *p* < 0.05.

## 3. Results

### 3.1. Descriptive Statistics and Correlations

Descriptive statistics, distributional characteristics, and bivariate correlations for PA and PSU across the three measurement waves are presented in Table 1. The PARS-3 composite scores showed moderate positive skewness across waves, with skewness values ranging from 1.42 to 1.60 and excess kurtosis values ranging from 1.65 to 2.44. Zero scores accounted for 16.95–17.83% of PA observations. Although the PA distributions deviated somewhat from normality, the observed skewness and kurtosis values did not indicate extreme non-normality. In contrast, PSU scores were approximately symmetric across waves, with skewness values of 0.14 and excess kurtosis values ranging from −0.59 to −0.47. PA was positively correlated across measurement waves (r = 0.231–0.408, all *p* < 0.01), as was PSU (r = 0.399–0.563, all *p* < 0.01). PA was negatively correlated with PSU both concurrently and across measurement occasions, with correlation coefficients ranging from −0.198 to −0.361 (all *p* < 0.01).

### 3.2. Longitudinal Measurement Invariance of the SAS-SV

Longitudinal measurement invariance was examined for the SAS-SV across T1, T2, and T3. As shown in Table 2, the configural, metric, and scalar invariance models all demonstrated good model fit. The configural model yielded CFI = 0.998, TLI = 0.998, RMSEA = 0.007, and SRMR = 0.023. Imposing equality constraints on factor loadings resulted in only minimal changes in model fit (ΔCFI = −0.001; ΔRMSEA = 0.002). Further constraining the item intercepts produced no meaningful deterioration in fit (ΔCFI = 0.000; ΔRMSEA = −0.001). These changes were well within the recommended criteria of |ΔCFI| ≤ 0.010 and |ΔRMSEA| ≤ 0.015, supporting configural, metric, and scalar invariance of the SAS-SV across the three measurement waves. Because the PARS-3 was treated as a composite exercise-volume index rather than a reflective latent construct, longitudinal measurement invariance based on a reflective factor model was not evaluated for the PARS-3.

### 3.3. Random-Intercept Cross-Lagged Panel Model

**Figure 2:**
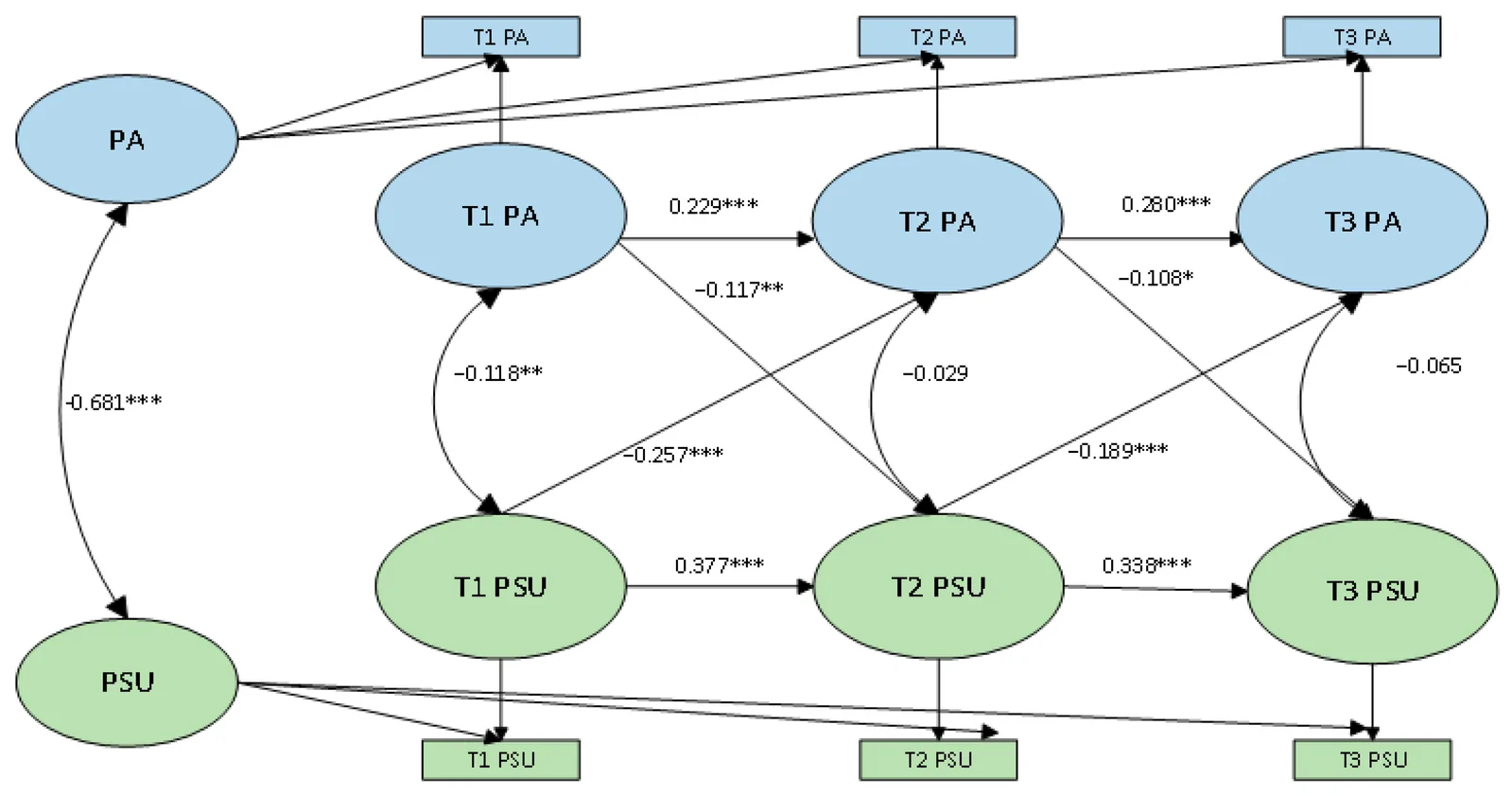
Standardized Path Coefficients of the Three-Wave Random-Intercept Cross-Lagged Panel Model Between PA and PSU. * *p* < 0.05, ** *p* < 0.01, *** p < 0.001.

The RI-CLPM was specified as the primary longitudinal model. The model converged normally and yielded χ^2^(1) = 0.033, *p* = 0.856, CFI = 1.000, TLI = 1.011, RMSEA = 0.000 (90% CI [0.000, 0.046]), and SRMR = 0.001. Given the single degree of freedom, the fit indices were interpreted cautiously. No negative freely estimated variance components were observed. Standardized path coefficients are presented in Figure 2, and full parameter estimates, including unstandardized coefficients, robust standard errors, and 95% confidence intervals, are reported in Table 3.

At the between-person level, the random intercepts of PA and PSU were negatively associated (β = −0.681, *p* = 0.004), indicating that adolescents with generally higher PA across the study period tended to report generally lower PSU. The random-intercept variances for both PA and PSU were statistically significant. At the within-person level, significant carry-over associations were observed for both PA and PSU across adjacent waves. For PA, the standardized autoregressive coefficients were 0.239 from T1 to T2 and 0.280 from T2 to T3; for PSU, the corresponding coefficients were 0.377 and 0.338, respectively.

Reciprocal cross-lagged associations were also observed across both six-month intervals. Higher-than-usual PSU was prospectively associated with lower-than-usual subsequent PA from T1 to T2 (β = −0.257, *p* < 0.001) and from T2 to T3 (β = −0.189, *p* < 0.001). Conversely, higher-than-usual PA was prospectively associated with lower-than-usual subsequent PSU from T1 to T2 (β = −0.117, *p* = 0.002) and from T2 to T3 (β = −0.108, *p* = 0.016). Detailed confidence intervals and unstandardized estimates are provided in Table 3. Within-wave associations between the time-specific PA and PSU components were small and were not consistently statistically significant across the three waves (Table 3). The proportions of explained within-person variance ranged from 13.1% to 13.8% for PA and from 13.8% to 16.6% for PSU.

### 3.4. Conventional Cross-Lagged Panel Model

**Figure 3:**
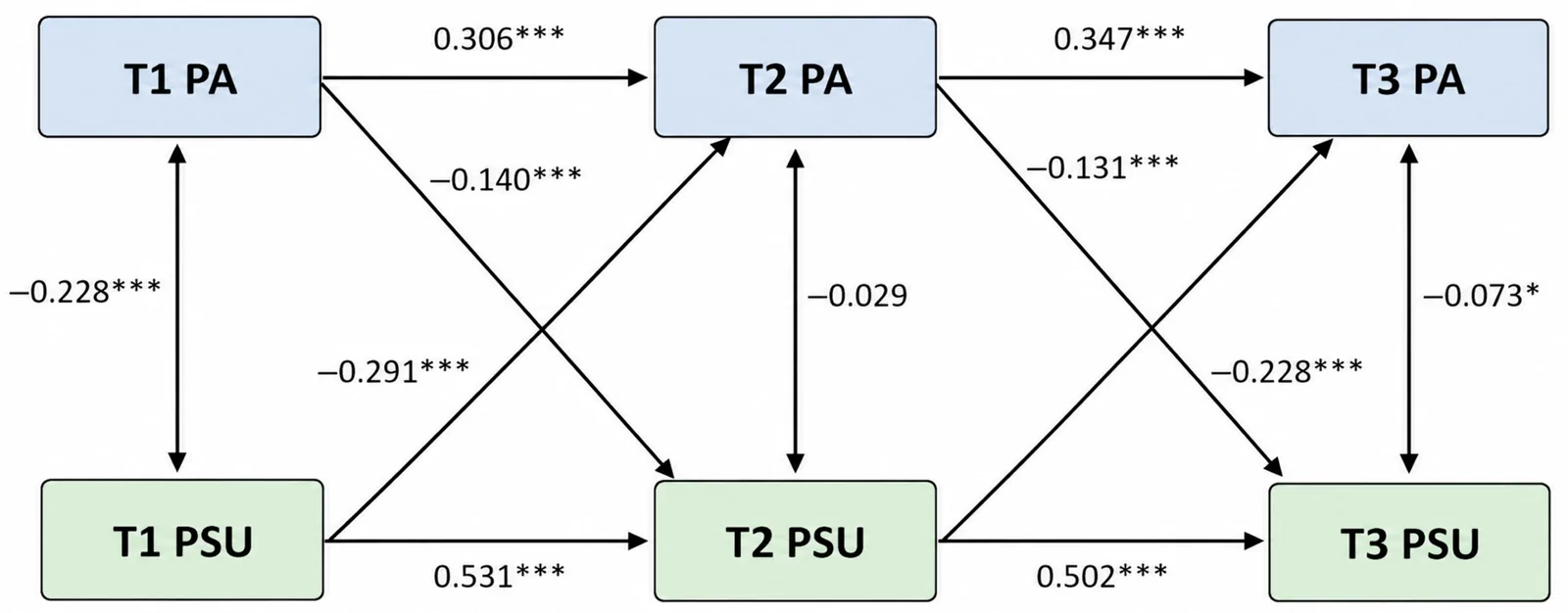
Standardized Path Coefficients of the Three-Wave Cross-Lagged Model Between PA and PSU. * *p* < 0.05, *** p < 0.001.

A conventional CLPM was estimated as a secondary analysis to facilitate comparison with previous longitudinal studies. The model showed adequate fit, χ^2^(4) = 14.121, *p* = 0.007, CFI = 0.992, TLI = 0.972, RMSEA = 0.050, and SRMR = 0.021. Standardized path coefficients are shown in Figure 3. Both PA and PSU showed significant autoregressive associations across adjacent waves. The autoregressive coefficients for PA were β = 0.306 from T1 to T2 and β = 0.347 from T2 to T3, whereas the corresponding coefficients for PSU were β = 0.531 and β = 0.502.

Reciprocal negative cross-lagged associations were observed across both intervals. Higher PA was associated with lower subsequent PSU from T1 to T2 (β = −0.140, *p* < 0.001) and from T2 to T3 (β = −0.131, *p* < 0.001). Higher PSU was likewise associated with lower subsequent PA from T1 to T2 (β = −0.291, *p* < 0.001) and from T2 to T3 (β = −0.228, *p* < 0.001).

## 4. Discussion

Using a three-wave longitudinal design, this study examined reciprocal prospective associations between PA and PSU among adolescents while distinguishing stable between-person differences from time-specific within-person deviations. At the between-person level, adolescents with generally higher PA across the study period tended to report lower overall PSU. At the within-person level, higher-than-usual PA was prospectively associated with lower-than-usual PSU at the subsequent wave, whereas higher-than-usual PSU was prospectively associated with lower-than-usual subsequent PA, with both patterns observed across the two six-month intervals. Significant carry-over associations were also observed for both PA and PSU. The secondary CLPM yielded reciprocal negative cross-lagged associations in the same directions.

### 4.1. Temporal Continuity of PA and PSU

The significant autoregressive effects observed for both PA and PSU indicate considerable behavioral continuity during adolescence. Adolescents who reported relatively high PA at one assessment tended to remain more active at the next assessment, whereas those with higher PSU were also likely to maintain elevated PSU over time. Such stability may reflect the habitual and context-dependent nature of both behaviors.

PA is shaped by relatively enduring factors, including exercise interest, self-efficacy, peer support, family encouragement, school-based opportunities, and daily schedules. Likewise, PSU may become increasingly habitual through repeated exposure to immediate digital rewards, social reinforcement, entertainment needs, and emotion-related coping (Hu et al., 2026d; Huang et al., 2026; Liu et al., 2026). Because self-regulatory capacities are still developing during adolescence, frequently repeated smartphone-use patterns may become particularly resistant to spontaneous change (Wan et al., 2025). These findings therefore suggest that prevention should begin before unhealthy behavioral routines become firmly established.

### 4.2. Prospective Association of PA with Subsequent PSU

Higher-than-usual PA was prospectively associated with lower-than-usual PSU at the subsequent assessment across both six-month intervals. This pattern is consistent with previous adolescent research reporting inverse prospective associations between PA and PSU (Xiao et al., 2022; Yoo, 2024). Zhao et al. (2024) for example, found that greater PA was associated with lower subsequent PSU and identified self-control as a potential explanatory pathway. The present study extends this literature by locating this prospective association at the within-person level after stable between-person differences were separated from time-specific deviations.

Time displacement provides one plausible explanation. Because adolescents have limited discretionary time, greater engagement in sports, outdoor recreation, and organized exercise may reduce opportunities for gaming, short-video viewing, social networking, and other smartphone-based activities (Lizandra et al., 2019). This account is consistent with the idea that PA and smartphone use compete, at least partly, for the same finite pool of leisure time (Röhlke, 2025; Sun et al., 2023).

Self-regulatory processes may provide an additional explanation. PA often involves planning, sustained effort, and goal-directed behavior, and previous research has linked exercise participation with inhibitory control and self-control among adolescents (Li et al., 2021). Regular engagement in PA may therefore coincide with behavioral patterns that facilitate greater regulation of smartphone use (Zhao et al., 2024; D. Zhang et al., 2022; Gao et al., 2024). PA may also provide alternative sources of enjoyment, social interaction, competence, and achievement, potentially reducing reliance on smartphone-based activities for similar psychological rewards.

The RI-CLPM further showed that occasions on which adolescents reported PA above their own expected level were followed by occasions characterized by lower-than-expected PSU. This finding distinguishes within-person fluctuations from the stable tendency for more active adolescents to report lower PSU than their peers. Nevertheless, the observed temporal ordering remains associational, and the present design cannot determine whether increasing PA itself produces subsequent reductions in PSU.

### 4.3. Prospective Association of PSU with Subsequent PA

Higher-than-usual PSU was prospectively associated with lower-than-usual PA at the subsequent assessment across both six-month intervals. This pattern suggests that the longitudinal relationship between the two behaviors is not confined to PA preceding subsequent PSU. It is also consistent with previous longitudinal and sensor-based research linking greater problematic or intensive smartphone use with lower subsequent exercise participation, fewer steps, and greater sedentary behavior among adolescents (Alexander et al., 2025; Burnell et al., 2026; Yu et al., 2025).

Time displacement provides one plausible explanation for this association. Extended engagement in short-video viewing, online gaming, social media, and other smartphone-based activities may compete with sports, outdoor recreation, and physically active social interaction for adolescents’ limited discretionary time. Objective monitoring studies similarly indicate that periods of greater smartphone use tend to coincide with fewer steps and less physical activity. Sleep-related disruption may further contribute to this pattern, as late-night smartphone use has been associated with shorter or delayed sleep and lower subsequent activity. Reduced sleep duration or irregular sleep schedules may, in turn, coincide with lower energy and fewer opportunities for sustained PA.

Behavioral regulation may provide an additional explanation. Repeated smartphone checking and rapid shifts in attention can interfere with planned activities, while highly accessible digital rewards may favor immediately reinforcing behaviors over activities that require greater initial effort. Previous studies have linked problematic smartphone use with poorer inhibitory control and greater sensitivity to immediate rewards (Deng et al., 2021; van Endert & Mohr, 2020; Gao et al., 2020; Telzer & Burnell, 2026). Such processes could make it more difficult for adolescents to initiate or maintain planned PA, although their specific contribution to the present longitudinal association remains to be established. The RI-CLPM further showed that occasions characterized by PSU above an adolescent’s own expected level were followed by occasions characterized by PA below that individual’s expected level. This within-person pattern indicates that the prospective association was observable even after stable differences between adolescents were separated from time-specific fluctuations.

### 4.4. Interpretation of the CLPM and RI-CLPM Findings

The conventional CLPM and RI-CLPM provide complementary but conceptually distinct perspectives on the longitudinal association between PA and PSU. In the CLPM, autoregressive and cross-lagged coefficients reflect adolescents’ relative standing over time but may combine stable between-person differences with within-person fluctuations. The CLPM was therefore retained primarily to facilitate comparison with previous longitudinal research rather than as an independent test of the within-person processes examined in the present study.

The RI-CLPM, which constituted the primary analytical model, separated stable between-person differences from time-specific within-person deviations. At the between-person level, the negative association between the random intercepts indicated that adolescents who were generally more physically active across the study period also tended to report lower overall PSU. At the within-person level, deviations from an adolescent’s own expected PA were prospectively associated with subsequent deviations in PSU, and the reverse pattern was also observed. These findings distinguish stable differences between adolescents from temporal fluctuations occurring within the same adolescent.

This distinction is important for interpreting the longitudinal findings. The autoregressive coefficients in the conventional CLPM primarily reflect rank-order stability, whereas those in the RI-CLPM represent carry-over in within-person deviations. Similarly, the cross-lagged paths from the two models should not be interpreted as estimates of the same underlying process. By separating these levels of variation, the RI-CLPM provides a more specific description of how PA and PSU covary prospectively within adolescents over time.

### 4.5. Theoretical Implications

The present findings refine the conceptual understanding of the PA–PSU relationship in adolescence in two important respects. First, they indicate that the inverse association between PA and PSU is not confined to stable differences between adolescents. Adolescents who were generally more active tended to report lower overall PSU, but deviations from an adolescent’s own typical level of one behavior were also prospectively associated with subsequent deviations in the other. This distinction is theoretically important because between-person differences and within-person dynamics address different questions: the former describe who is generally more active or more prone to PSU, whereas the latter concern how changes relative to an individual’s own behavioral baseline are linked over time. Treating these two levels as interchangeable may obscure the processes through which PA and PSU become coupled during adolescence.

Second, the reciprocal within-person pattern suggests that PA and PSU may be better conceptualized as dynamically interrelated components of adolescents’ daily behavioral organization rather than as a simple predictor–outcome pair. Existing research has often emphasized PA as an antecedent of lower PSU, but the present findings indicate that fluctuations in PSU may also precede subsequent fluctuations in PA. This reciprocal structure is compatible with theoretical accounts centered on competition for discretionary time, behavioral self-regulation, and reinforcement processes. Importantly, such mechanisms may operate in both directions: greater engagement in one behavior may alter the temporal, motivational, or regulatory conditions under which the other occurs. A bidirectional framework therefore provides a more comprehensive basis for explaining how active and digitally oriented behaviors may become organized within adolescents’ everyday lives.

The separation of between-person and within-person variance also has implications for future theory development. Stable individual differences may reflect relatively enduring social, motivational, or environmental conditions, whereas within-person fluctuations may be more sensitive to short-term changes in school demands, leisure opportunities, peer interaction, sleep routines, or digital engagement. Future longitudinal research should therefore move beyond testing whether PA and PSU are associated and examine when, for whom, and under what contextual conditions their within-person coupling becomes stronger or weaker. Incorporating time-varying explanatory variables and more intensive measurement designs would allow theoretical models to specify the processes linking these behaviors with greater temporal precision.

### 4.6. Implications for Prevention and Intervention Research

The findings have implications less for prescribing a specific intervention than for how future adolescent health interventions should be conceptualized and evaluated. PA and PSU are commonly addressed within separate research and prevention traditions, yet the present within-person findings indicate that the two behaviors may change in relation to one another over time. Intervention studies may therefore benefit from treating PA and PSU as interconnected behavioral outcomes, even when only one is the primary target. For example, programs designed to increase PA could assess whether changes in activity are accompanied by changes in smartphone-use patterns, while interventions targeting PSU could evaluate whether reductions in problematic use coincide with changes in subsequent activity.

The results also point toward the importance of behavioral context rather than isolated exposure reduction. Simply increasing opportunities for exercise or imposing restrictions on smartphone use may overlook the daily routines within which both behaviors occur. School-based and family-based programs could instead examine how leisure time is structured, when smartphone use displaces active behavior, and whether physically active alternatives provide sufficiently accessible and rewarding options. This perspective shifts attention from treating PA and PSU as competing health messages to understanding how adolescents allocate time, attention, and behavioral resources across the day.

A further implication concerns intervention timing and personalization. The within-person results indicate that departures from an adolescent’s usual behavioral pattern may contain information that is not captured by between-person risk classification alone. Future studies using ecological momentary assessment, passive smartphone sensing, or wearable activity monitoring could determine whether short-term increases in PSU or declines in PA identify periods during which behavioral support may be particularly relevant. Such designs would also allow researchers to test whether interventions that respond to individual behavioral fluctuations are more effective than approaches based solely on static risk categories.

### 4.7. Limitations and Future Directions

Several limitations should be considered when interpreting the present findings. First, both PA and PSU were assessed using self-report measures. The PARS-3 provides a composite index of exercise intensity, duration, and frequency and therefore captures exercise-related activity more directly than total daily movement. Its scores were also moderately positively skewed, with approximately 17% of participants reporting a score of zero across waves. Although robust maximum-likelihood estimation was used, future studies would benefit from combining self-reported exercise with accelerometry and objective smartphone-use indicators, such as screen-time and application-use records. Such multimethod assessment would reduce shared measurement bias and allow the reciprocal associations to be evaluated across different operationalizations of both behaviors.

Second, the analyses were based on the 1015 adolescents who provided successfully matched data across all three waves, representing a cumulative attrition of 19.06% from baseline. Using the same cohort across measurement occasions ensured that the longitudinal models were estimated from an identical set of participants; however, complete-case analysis necessarily discarded information from participants with incomplete follow-up and may have reduced precision or introduced selection bias. Baseline comparisons between retained and lost participants can characterize observable differences but cannot establish that missingness was completely random. Future replications using all available observations through FIML or other principled missing-data approaches would provide an important test of the sensitivity of the present estimates to attrition.

Third, the school-based sampling structure and developmental transitions across the follow-up period were only partially represented in the models. Participants were recruited from six schools and were nested within shared educational environments, yet school- and class-level dependence was not explicitly modeled. In addition, students recruited in Grades 8 and 11 subsequently entered Grades 9 and 12, respectively, meaning that some participants experienced examination-year transitions during follow-up. Such transitions may coincide with changes in academic demands, opportunities for PA, and patterns of smartphone use. Sex, age, and grade were also not modeled as potential sources of heterogeneity. Studies with a larger number of schools and classes should examine whether the within-person associations vary across educational contexts and developmental groups, including through multilevel or moderated longitudinal models.

Fourth, three assessments separated by approximately six months provide only one temporal resolution of the PA–PSU relationship. The processes linking the two behaviors may operate over markedly different timescales: competition for discretionary time or smartphone checking may occur within the same day, whereas changes in exercise routines or problematic use may develop over weeks or months. With only three waves, the present study was also limited in its ability to examine more complex temporal dynamics or determine whether longitudinal parameters are invariant across intervals. More intensive designs—including daily diaries, ecological momentary assessment, passive smartphone sensing, and repeated wearable-based PA assessment—could clarify the timescale at which within-person coupling between PA and PSU is most pronounced.

Fifth, the RI-CLPM separates stable between-person differences from time-specific within-person deviations, but it does not eliminate time-varying confounding or establish causal effects. Several potentially important factors were not assessed in the present study, including sleep quality, depressive symptoms, academic stress, parental monitoring, peer relationships, physical health, family socioeconomic conditions, and the specific functions for which adolescents use smartphones. Changes in these factors could plausibly precede or accompany changes in both PA and PSU. Future research should therefore incorporate theoretically relevant time-varying variables and examine whether the observed reciprocal associations persist after these processes are modeled explicitly. Experimental and intervention studies would ultimately be needed to determine whether deliberately changing one behavior produces subsequent change in the other.

Finally, participants were recruited through convenience sampling from six secondary schools in Jiangxi Province. The extent to which the present between-person and within-person associations generalize to adolescents from other regions, school systems, socioeconomic backgrounds, or cultural settings remains uncertain. Replication in geographically and socially diverse samples will be important for establishing the broader generalizability of the findings.

## 5. Conclusions

Using a three-wave longitudinal design, this study examined the reciprocal prospective associations between PA and PSU among adolescents, with the RI-CLPM serving as the primary analytical framework. At the between-person level, adolescents with generally higher PA across the study period tended to report lower overall PSU. At the within-person level, higher-than-usual PA was prospectively associated with lower-than-usual subsequent PSU, whereas higher-than-usual PSU was prospectively associated with lower-than-usual subsequent PA across both six-month intervals. These findings extend previous research by showing that the PA–PSU relationship involves not only stable differences between adolescents but also time-specific fluctuations within the same individual. From an applied perspective, the results support considering PA and problematic smartphone use jointly when developing adolescent health-promotion strategies, while further experimental and intervention research is needed to determine whether modifying one behavior leads to subsequent changes in the other.

## Figures and Tables

**Table 1:** Descriptive Statistics, Distributional Characteristics, and Bivariate Correlations for PA and PSU Across Three Waves.

Variable	M	SD	Skewness	Kurtosis	Zero Scores, %	1	2	3	4	5	6
1. T1 PA	19.43	22	1.6	2.44	17.83	1					
2. T2 PA	19.92	21.89	1.49	1.92	17.44	0.373 **	1				
3. T3 PA	20.2	22.1	1.42	1.65	16.95	0.231 **	0.408 **	1			
4. T1 PSU	32.68	9.8	0.14	−0.59	/	−0.228 **	−0.361 **	−0.259 **	1		
5. T2 PSU	32.66	9.79	0.14	−0.57	/	−0.262 **	−0.265 **	−0.320 **	0.563 **	1	
6. T3 PSU	32.7	9.81	0.14	−0.47	/	−0.198 **	−0.264 **	−0.268 **	0.399 **	0.537 **	1

Note: PA = physical activity; PSU = problematic smartphone use. Kurtosis values represent excess kurtosis, for which a normal distribution has a value of 0. Zero scores indicate participants with a PARS-3 composite score of 0 and are not applicable to the SAS-SV. ** *p* < 0.01.

**Table 2:** Longitudinal Measurement Invariance of the SAS-SV Across Three Waves.

Scale	Model	χ^2^	df	CFI	TLI	RMSEA	SRMR	ΔCFI	ΔRMSEA
SAS-SV	Configural invariance	420.09	402	0.998	0.998	0.007	0.023	—	—
	Metric invariance	451.03	420	0.997	0.997	0.009	0.025	−0.001	0.002
	Scalar invariance	464.72	438	0.997	0.997	0.008	0.025	0	−0.001

**Table 3:** Parameter Estimates for the Three-Wave Random-Intercept Cross-Lagged Panel Model Between PA and PSU.

Parameter	B	SE	95% CI for B	β	95% CI for β	*p*
Autoregressive paths						
T1 PA → T2 PA	0.238	0.061	[0.118, 0.358]	0.239	[0.121, 0.358]	<0.001
T2 PA → T3 PA	0.283	0.057	[0.170, 0.395]	0.28	[0.167, 0.392]	<0.001
T1 PSU → T2 PSU	0.377	0.054	[0.271, 0.483]	0.377	[0.276, 0.478]	<0.001
T2 PSU → T3 PSU	0.338	0.055	[0.230, 0.446]	0.338	[0.230, 0.445]	<0.001
Cross-lagged paths						
T1 PA → T2 PSU	−0.047	0.016	[−0.078, −0.017]	−0.117	[−0.191, −0.042]	0.003
T2 PA → T3 PSU	−0.044	0.018	[−0.080, −0.009]	−0.108	[−0.196, −0.020]	0.014
T1 PSU → T2 PA	−0.629	0.121	[−0.867, −0.391]	−0.257	[−0.346, −0.169]	<0.001
T2 PSU → T3 PA	−0.467	0.109	[−0.680, −0.254]	−0.189	[−0.273, −0.104]	<0.001
Between-person association						
RI-PA ↔ RI-PSU	−29.160	10.177	[−49.107, −9.214]	−0.681	[−0.977, −0.385]	0.004

Note. →regression effect, and ↔ indicates a bidirectional association between variables.

## Data Availability

The data presented in this study are available from the corresponding author upon reasonable request. The data are not publicly available because they contain information collected from adolescent participants and are subject to ethical and privacy restrictions.

## Supplementary Materials

The following supporting information can be downloaded at: https://www.mdpi.com/article/10.3390/bs16091557/s1, Table S1. Comparison of baseline demographic and study characteristics between three-wave completers and participants with incomplete follow-up.
