# Supplementary material for: The Relationship Between Physical Activity and Mobile Phone Addiction Among Adolescents: A Cross-Lagged Study
Source: Behav Sci (Basel). 2026 Sep 2;16(9):1557. doi: 10.3390/bs16091557 (PMC13603538; doi:10.3390/bs16091557)
Supplement: Supplementary file 1 [file behavsci-16-01557-s001.zip › behavsci-4492999-supplementary.pdf]

Table S1. Comparison of baseline demographic and study characteristics between three-wave completers and participants with incomplete follow-up

Characteristics	Three-wave completers (n=1015)	Incomplete follow-up (n=239)	Effect size	P value
Sex, n (%)			Cramér's V=0.011	0.697
Male	425 (41.9)	104 (43.5)		
Female	590 (58.1)	135 (56.5)		
Grade, n (%)			Cramér's V=0.018	0.935
Grade 7	280 (27.6)	61 (25.5)		
Grade 8	270 (26.6)	65 (27.2)		
Grade 10	245 (24.1)	59 (24.7)		
Grade 11	220 (21.7)	54 (22.6)		
Residence, n (%)			Cramér's V=0.011	0.701
Rural	493 (48.6)	120 (50.2)		
Urban	522 (51.4)	119 (49.8)		
Only child, n (%)			Cramér's V=0.012	0.684
Yes	305 (30.0)	68 (28.5)		
No	710 (70.0)	171 (71.5)		
Father's education, n (%)			Cramér's V=0.011	0.997
Primary school or below	150 (14.8)	37 (15.5)		
Junior high school	331 (32.6)	79 (33.1)		
High school/technical school	256 (25.2)	60 (25.1)		
College	162 (16.0)	37 (15.5)		
Bachelor's degree or above	116 (11.4)	26 (10.9)		
Mother's education, n (%)			Cramér's V=0.007	>0.999
Primary school or below	168 (16.6)	41 (17.2)		
Junior high school	330 (32.5)	77 (32.2)		
High school/technical school	280 (27.6)	65 (27.2)		
College	144 (14.2)	34 (14.2)		
Bachelor's degree or above	93 (9.2)	22 (9.2)		
Monthly family income, n (%)			Cramér's V=0.007	0.969
<3000 RMB	331 (32.6)	77 (32.2)		
3000–6000 RMB	437 (43.1)	105 (43.9)		
>6000 RMB	247 (24.3)	57 (23.8)		
Age, years, M (SD)	14.86 (1.61)	14.92 (1.57)	SMD=−0.037	0.597
PA at T1, M (SD)	19.43	20.60	SMD=−0.053	0.473

	(22.00)	(22.80)		
PSU at T1, M (SD)	32.68 (9.80)	33.10 (10.15)	SMD=−0.043	0.563
